# 

**Published:** 1888-10-27

**Authors:** 


### CONTENTS.

**Diphtheria in London** - - - - 49

**King's College Hospital Revisited** - - - - 50

**The Doctor's Pulpit.**—The Seven Ages of Man—IV. The Soldier - 51

**Australasian Science Association** - - - 52

**The Odd Fellows' Convalescent Scheme** - - - 52

**Town Smoke and Fogs.**—By DAVID WALSH, L.R.C.P. and S. Edin. - 53

**Voyages in Vacation.**—V. Companions and Entertainments - 55

**Notes and News** - - - - - - 56

**Everybody's Page** - - - - - 58

**Annotations.**—Lady Guardians.—Murder and "Penny Dreadfuls."—The Tyrannies of the Workshop - 59

**Physiology and its Uses.**—The Blood - - - 60

**Turning Over New Leaves.**—"Medicine and Surgery at Guy's Hospital."—"Foster's Physiology." - 61

**The Student's Column** - - - - 62

**False Impressions.** By CAROLINE FOTHERGILL, Author of "An Enthusiast," "A Voice in the Wilderness," etc. - 63

**Amusements and Relaxation** - - - 64

**EXTRA SUPPLEMENT.—THE NURSING MIRROR.**

**En Passant** — Florence Nightingale. — Obstructing a Monthly Nurse.—The British Nurses' Association.—Emigration.—Fifty Pounds Wanted.—A Memorial to Mrs. Bromhead.—The Empress Frederick as Nurse - - - - - xiii

**Appointments, Lectures, Vacancies, Notes & Queries** xv

**Facts for Nurses** Concerning the General Structure of the Human Body - - - - - xiv

**Everybody's Opinion.**—A Convalescent Home for Nurses.—The British Nurses' Association.—The Private Nurse.—A Melbourne Hospital - - - - - - xv

